# Comparison of common pharmacological strategies for post-photorefractive keratectomy pain management: a systematic review and network meta-analysis

**DOI:** 10.3389/fmed.2026.1904962

**Published:** 2026-08-10

**Authors:** Xin Yan, Shiqi Lei, Jiasheng Yi, Lifen Hu, Mu Qin

**Affiliations:** Department of Ophthalmology, The Affiliated Hospital (Clinical College) of Xiangnan University, Xiangnan University, Chenzhou, China

**Keywords:** comparative effectiveness, drug therapy, network meta-analysis, photorefractive keratectomy, safety

## Abstract

**Objective:**

To systematically evaluate the comparative efficacy and safety of common pharmacological strategies for managing post-photorefractive keratectomy (PRK) eye pain through a network meta-analysis, providing evidence-based guidance for clinical medication.

**Methods:**

Randomized controlled trials (RCTs) were systematically retrieved from Cochrane Library, Web of Science, PubMed, and EMbase (from inception to November 15, 2025). Using R Studio, we conducted a network meta-analysis to assess differences in Visual Analogue Scale (VAS) scores and adverse events across pharmacological interventions. SUCRA (Surface Under the Cumulative Ranking) values were calculated to determine the relative ranking of interventions, while accounting for heterogeneities in administration timing and dosage.

**Results:**

A total of 28 studies were included, involving 2,797 patients. In route-stratified analyses, Ofloxacin (SUCRA = 0.84) and Proparacaine (0.76) were the top-ranked topical agents for analgesia, while Codeine combined with Acetaminophen (0.71) ranked highest among systemic agents. The exploratory combined network yielded identical top-three rankings, though these cross-route comparisons are susceptible to transitivity violations. Notably, effect sizes for most interventions relative to placebo spanned the line of no effect. Regarding safety, Diclofenac (SUCRA = 0.81), Ketorolac (0.70), and Nepafenac (0.53) demonstrated the optimal profiles. The highest numbers of adverse events occurred with Pregabalin (39 cases), Fentanyl (20 cases), and Codeine combined with Acetaminophen (13 cases); however, safety conclusions remain limited as only seven studies reported highly heterogeneous adverse event data.

**Conclusion:**

Ofloxacin demonstrates superior relative efficacy for pain control following PRK, while Diclofenac offers the most favorable safety profile. Clinicians should synthesize these comparative findings with individual patient profiles to optimize drug selection, ensuring a balanced approach between effective pain relief and minimized adverse risks.

**Systematic review registration:**

https://www.crd.york.ac.uk/PROSPERO/view/CRD420251238092, CRD420251238092.

## 1 Introduction

Postoperative ocular pain following photorefractive keratectomy (PRK) is a common and clinically significant complication that adversely affects patient recovery and satisfaction. The incidence of post-PRK pain is high, with peak intensity typically occurring within the first 24–72 h after surgery (1). While most patients experience resolution of pain within the acute phase, a non-negligible subset—approximately 11% in prospective cohorts—report persistent pain extending to 3–6 months postoperatively, suggesting that acute postoperative pain may, in some individuals, transition to a chronic pain state (1, 2). This chronicity is multifactorial, with preoperative ocular pain, depressive symptoms, and concomitant anti-allergy medication use identified as independent risk factors (2). The existence of this chronic pain phenotype underscores the need for not only effective acute analgesia but also strategies that may mitigate the risk of pain persistence—a goal that requires a nuanced understanding of comparative drug performance across different pain mechanisms.

Current clinical practice for post-PRK pain management employs a combination of topical and systemic pharmacological agents spanning multiple drug classes. Topical non-steroidal anti-inflammatory drugs (NSAIDs), including ketorolac, flurbiprofen, diclofenac, and nepafenac, are widely used to inhibit prostaglandin synthesis and provide local analgesia; however, their efficacy varies across agents, and concerns regarding delayed corneal epithelial healing and, in rare cases, corneal melting—particularly in patients with autoimmune conditions—have been raised (3, 4). Opioid analgesics, such as codeine combined with acetaminophen, offer systemic pain relief but are associated with dose-dependent adverse effects including nausea, drowsiness, and constipation, and their controlled substance status limits accessibility in some regions (5). Neuromodulators, including gabapentin and pregabalin, have demonstrated efficacy in attenuating acute postoperative pain, likely through modulation of central sensitization, yet their long-term safety profile and impact on dry eye symptoms remain incompletely characterized (6, 7). Topical anesthetics like proparacaine and lidocaine provide rapid-onset analgesia but carry risks of corneal toxicity and rebound pain upon discontinuation (8). Emerging agents, such as the TRPV1 antagonist SAF312 (Libvatrep), offer a novel mechanism targeting nociceptive pathways without impairing wound healing, while topical nalbuphine—an opioid agonist–antagonist—has shown dose-dependent analgesic effects in early postoperative periods (9, 10). Despite this pharmacological armamentarium, the diversity of mechanisms and safety profiles poses a challenge for clinicians attempting to select an optimal regimen.

Several limitations in the existing evidence base further complicate clinical decision-making. First, the vast majority of randomized controlled trials (RCTs) adopt a single-drug-versus-placebo design, providing no direct head-to-head comparisons between active agents. This paucity of direct comparative data leaves clinicians without empirical guidance on the relative efficacy or safety of one drug over another. Second, safety concerns are drug-specific and class-dependent: NSAIDs may impair epithelial healing; opioids cause systemic adverse effects; local anesthetics carry toxicity risks; and neuromodulators have uncertain long-term effects. These divergent safety profiles cannot be meaningfully weighed against each other without a unified comparative framework. Third, existing systematic reviews have largely focused on individual drug classes—such as NSAIDs alone (4) or pregabalin alone (6, 7)—and have not attempted to integrate evidence across pharmacologically distinct categories. Consequently, no study to date has provided a comprehensive, head-to-head comparison of the relative efficacy and safety of antibiotics, local anesthetics, opioids, neuromodulators, and NSAIDs within a single analytic framework.

Network meta-analysis offers a robust methodological solution to these gaps by synthesizing direct and indirect evidence from placebo-controlled trials, enabling simultaneous comparison and ranking of multiple interventions. This approach not only overcomes the lack of head-to-head RCTs but also allows for a quantitative integration of efficacy and safety outcomes across drug classes. By constructing a comparative network, we can generate clinically actionable rankings—while acknowledging the exploratory nature of such rankings—and identify agents that may offer the most favorable balance between pain relief and tolerability. Moreover, the ability to rank interventions provides a foundation for future individualized treatment strategies, wherein drug selection could be tailored to patient-specific risk factors (e.g., autoimmune status, baseline pain sensitivity, or dry eye propensity). Therefore, the present study aims to conduct a network meta-analysis of RCTs to systematically compare the relative efficacy and safety of commonly used drugs for post-PRK ocular pain, with the goal of informing evidence-based, patient-centered clinical decision-making.

## 2 Methods

### 2.1 Registration

This study was conducted in accordance with PRISMA’s requirements. We have registered with PROSPERO.^1^

### 2.2 Inclusion criteria

Subjects: Patients with eye pain after Photorefractive Keratectomy (PRK), regardless of nationality, ethnicity, age, gender, or language. For PRK subtypes, both traditional PRK and transepithelial PRK (t-PRK) were included, provided that the intervention under evaluation was a pharmacological agent for postoperative pain management. This decision was made because both procedures are surface ablation techniques with comparable postoperative pain mechanisms, and excluding t-PRK would have substantially reduced the available evidence base.

Interventions: Eligible interventions included any pharmacological agent evaluated for postoperative pain management after PRK, encompassing the following drug classes: NSAIDs, opioids, neuromodulators, NMDA receptor antagonists, local anesthetics, antibiotics (ofloxacin), corticosteroids, alpha-2 agonists, and nalbuphine.

Given the substantial heterogeneity in mechanisms of action and administration routes among these agents, we adopted a hierarchical analytic approach. For the primary analysis, we constructed separate networks stratified by administration route: topical agents (including ophthalmic solutions, drops, and drug-soaked bandage contact lenses) and systemic agents (including oral, transdermal, and parenteral formulations). For the secondary analysis, we pooled all interventions into a combined network as a sensitivity analysis, with results interpreted as exploratory. This strategy allows clinically meaningful comparisons within homogeneous classes while offering a comprehensive evidence overview. Different administration routes (e.g., topical vs. oral NSAIDs) were not combined in the primary analyses but were retained in separate networks; they were only pooled in the exploratory sensitivity analysis.

*Control group*: The drug or placebo of the experimental group.

*Outcome measure*: Visual Analog Scales (VAS) score (11); Adverse reactions (AE).

*Research type*: Randomized controlled trial.

### 2.3 Exclusion criteria

Duplicate publications; reviews, animal experiments, case reports, or other non-RCT study designs; studies with no relevant outcome indicators or from which valid data could not be extracted; studies reporting fewer than two interventions for the same outcome, rendering network meta-analysis infeasible due to the absence of a connected network structure.

### 2.4 Literature search strategy

The Cochrane Library, Web of Science, PubMed, and EMbase databases were searched by computer until November 15, 2025. Searches were conducted using a combination of subject terms and free terms, and references to relevant literature were manually retrieved. Search strategies are provided in Supplementary Table S1.

### 2.5 Literature screening and data extraction

Two researchers independently screened the literature and extracted the data strictly in accordance with the inclusion and exclusion criteria, cross-checked them, discussed or consulted a third party in case of disagreement, and reached a consensus. The extracted content included first author, publication year, sample size, intervention measures, control group, intervention time, outcome measure.

### 2.6 Risk of bias assessment

Quality assessment and risk of bias assessment of the included literature were conducted using ROB2 (12), the risk of bias assessment tool recommended by Cochrane Hand book5.3.0. RoB2 set up five evaluation domains: bias during randomization, bias from established interventions, bias due to missing outcome data, bias in outcome measurement, and bias in selective reporting of results. The risk of bias in each domain can be classified into three levels: low risk, unknown risk, and high risk. Two researchers independently evaluate and compare the results. If there is disagreement, consult a third researcher or discuss and decide.

### 2.7 Statistical analysis

Statistical analyses were performed using R studio (gemtc package) within a Bayesian framework. A random-effects model was consistently applied across all network meta-analyses, as it accounts for both within-study and between-study variability and is generally preferred in NMA given the anticipated clinical and methodological diversity across included studies. Non-informative uniform priors (dunif (0, 5) for between-study standard deviation) were chosen to minimize the influence of prior beliefs on posterior estimates. The transitivity assumption was assessed by comparing the distributions of potential effect modifiers across treatment comparisons, including baseline pain intensity, concomitant medications, surgical technique, and timing of outcome assessment. When systematic imbalances were detected, affected comparisons were flagged as exploratory. Four MCMC chains were run with 50,000 iterations each, including a burn-in of 20,000 iterations. Convergence was assessed using PSRF, with PSRF <1.05 considered acceptable. Binary outcomes were reported as RR, continuous outcomes as MD, both with 95% CrI. To address clinical heterogeneity in pain assessment timing, VAS scores at 24 h post-surgery (or nearest available time point) were extracted; if studies reported multiple time points, the average of the first 3 postoperative days was used. In primary analyses, different administration routes were assigned to separate networks (topical vs. systemic) to preserve transitivity; the combined network (pooling all routes) was reserved as an exploratory sensitivity analysis. Global consistency was evaluated using both the design-by-treatment interaction model (testing overall inconsistency across the entire network) and the node-splitting method (testing local inconsistency for each comparison with both direct and indirect evidence). A non-significant result in either test (*p* > 0.05) was interpreted as supporting the consistency assumption. Multi-arm trials were handled by accounting for correlations between effect estimates from multiple comparisons within the same study, using the multivariate normal likelihood with a compound symmetry structure as implemented in the gemtc package. For the combined network, we additionally performed a sensitivity analysis excluding multi-arm trials to assess the robustness of the rankings. Heterogeneity was quantified using the Cochrane *Q* test and *I*^2^ statistic to describe the degree of between-study variation; however, model choice was not determined by these metrics, as the random-effects model was applied consistently. To explore potential heterogeneity sources, we conducted sensitivity analyses by sequentially excluding studies with outlying effect estimates or those with high risk of bias; Treatment rankings were derived from SUCRA values. Publication bias was evaluated using comparison-adjusted funnel plots and Egger’s test (Stata 16).

### 2.8 Quality of evidence assessment

We used CINeMA (13) to assess the quality of evidence. CINeMA included six areas: intra-study bias, inter-study bias, indirectness, imprecision, heterogeneity, and inconsistency, to grade the quality of evidence. Ultimately, the quality of the evidence is classified into four grades: high, medium, low, and very low quality.

## 3 Results

### 3.1 Study selection

**Figure 1:**
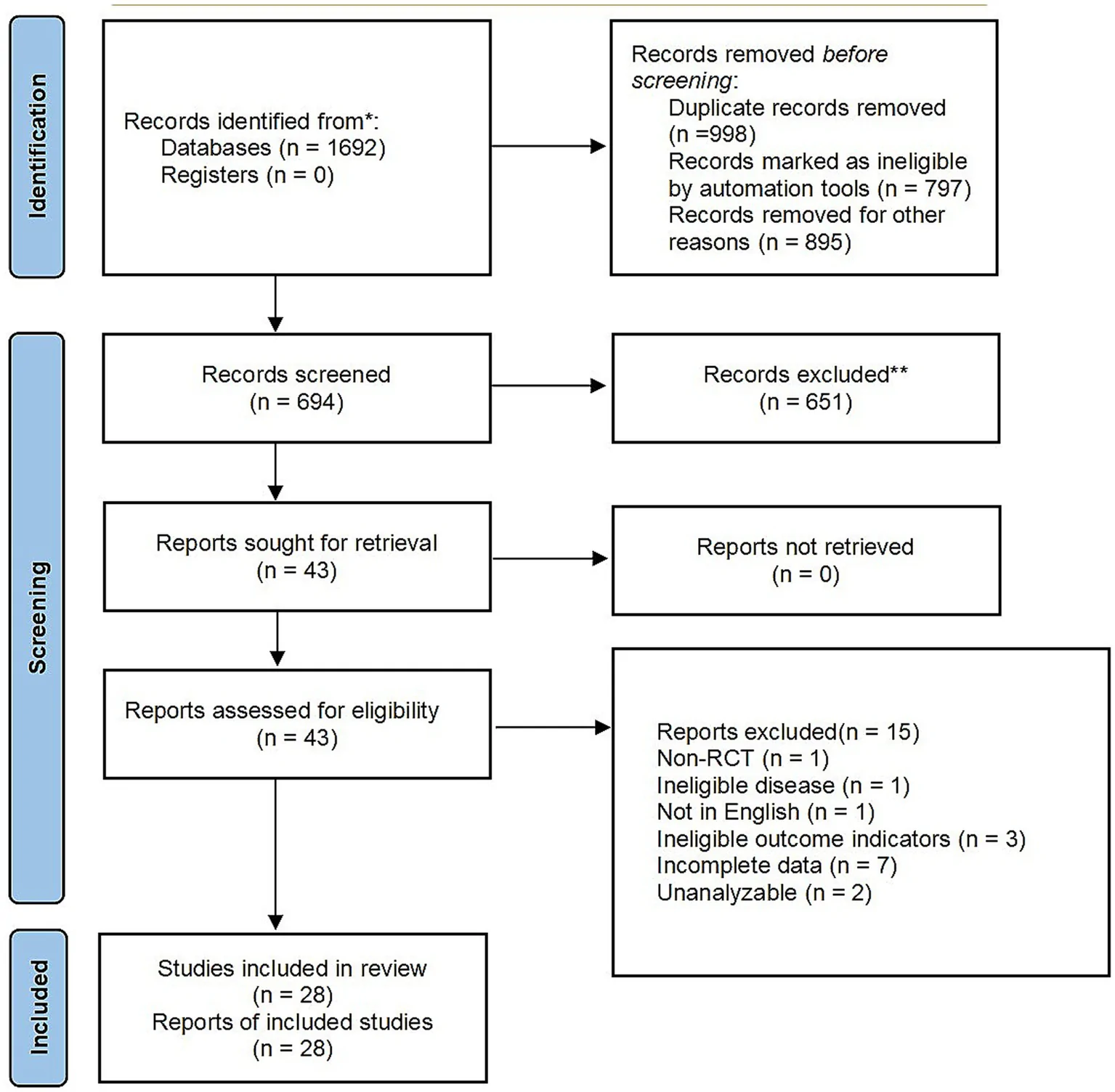
Literature screening process. *Consider, if feasible to do so, reporting the number of records identified from each database or register searched (rather than the total number across all databases/registers). **If automation tools were used, indicate how many records were excluded by a human and how many were excluded by automation tools.

Preliminary search yielded 1,692 relevant documents, 998 duplicate documents were excluded, 651 were excluded by reading titles and abstracts, and 15 were excluded by reading full texts. Eventually, 28 studies were included. Figure 1 shows the screening flowchart.

### 3.2 Study characteristics

This study included 28 RCTs (5, 14–40) with a total of 2,797 patients, evaluating 14 drugs across five pharmacological classes: NSAIDs, topical anesthetics, opioids, neuromodulators, and quinolone antibiotics. Regarding PRK subtypes, the included trials covered conventional PRK, bilateral PRK, alcohol-assisted PRK (aaPRK), and one transepithelial PRK (t-PRK) study (40). Interventions were administered via multiple routes (topical, oral, and transdermal), with timing ranging from preoperative prophylaxis to postoperative rescue analgesia; follow-up durations varied from 1 to 7 days. The majority of studies reported VAS as the primary efficacy outcome, while seven studies provided adverse event data. Geographically, the studies were predominantly from North America and the Middle East, with limited representation from other regions (Table 1). This geographic concentration should be considered when generalizing the findings to broader populations with differing clinical practices or drug access.

**Table 1:** Basic characteristics of included studies.

Study	Year	Types of PRK	Country	Sample size	Types of drugs	Intervening measure(T/C)	Time	Outcomes
Miles K. Tutton (14)	1996	mPRK	Switzerland	25/25	NSAIDs	Diclofenac0.1%/ Placebo	Every 2–5 h (from 60 min pre-op)	VAS
V. Moss Weinstock (15)	1996	Bilateral mPRK	Canada	51/51	NSAIDs	Diclofenac0.1%/ Ketorolac0.5%	Every 4 h	VAS
Lee Shahinian (16)	1997	PRK	California	34	Ester local anesthetics	Proparacaine0.05%/Placebo	7d	VAS
Michael Assouline (17)	1998	Unilateral PRK	France	40/40/40	NSAIDs	Indomethacin0.1%/ Diclofenac0.1%/ Placebo	4/d	VAS
Appiotti (18)	1998	Bilateral PRK	Italy	9/7	NSAIDs	Flurbiprofen0.03%/Diclofenac0.1%	6/d for 3 days	VAS
Rajesh K. Rajpal (19)	1999	First-eye PRK	USA	102/98	NSAIDs	Ketorolac0.5%/ Placebo	4/d for 3 days	AE
Kerry D. Solomon (20)	2004	Unilateral PRK	USA	156/157	NSAIDs	Ketorolac0.4%/ Placebo	4/d up to 4 days	AE
Joseph Colin (21)	2006	PRK	France	20/20	NSAIDs	Nepafenac0.1%/ Diclofenac0.1%	4/d for 7 days	AE
Eric D. Donnenfeld (22)	2007	Bilateral mPRK	USA	38	NSAIDs	Nepafenac0.1%/ Ketorolac0.4%	3/d for 3 days	VAS, AE
Daniel S. Durrie (23)	2007	aaPRK	USA	30/16/12	NSAIDs	Nepafenac0.1%/ Ketorolac0.4%/ Bromfenac0.09%	3/d	VAS
Matthew Caldwell (24)	2008	Bilateral aaPRK	USA	33/33	NSAIDs	Nepafenac0.1%/ Placebo	3d	VAS
Neal A. Sher (25)	2009	PRK	USA	149	NSAIDs	Bromfenac0.09%/ Ketorolac0.4%	2/d 4/d	VAS
Ella G. Faktorovich (26)	2010	PRK	California	20/20	Opioid	Morphine0.5%/Placebo	4/d for 7 days	VAS
Mehrdad Mohammadpour (27)	2011	Bilateral aaPRK	Iran	70/70	NSAIDs	Diclofenac0.1%/ Placebo	1 drop 30 min pre-op	VAS
Matthew D. Kuhnle (28)	2011	PRK	USA	41/41	Neuroregulator	Gabapentin/Placebo	4d	VAS
Hassan Razmju (29)	2012	Bilateral aaPRK	Iran	47/36	NSAIDs	Diclofenac0.1%/ Ketorolac0.5%/ Placebo	2d	VAS
Mohammad Pakravan (30)	2012	PRK	Iran	50/50/50	Neuroregulator	Pregabalin0.07%/Gabapentin0.3%/Placebo	3d	VAS
Alireza Eslampour (31)	2013	PRK	Iran	33/29	NSAIDs	Diclofenac0.1%/Acetaminophen0.4% + Ibuprofen0.4%	4/d for 6 days	VAS
Alireza Eslampoor (32)	2014	Bilateral PRK	Iran	38/30	NSAIDs	Diclofenac0.1%/Placebo	4/d for 3 days	VAS
Jin Pyo Hong (33)	2014	Bilateral aaPRK	South Korea	49/45	NSAIDs	Ketorolac0.5%/Ofloxacin0.3%/ Diclofenac0.1%		VAS
Julie M. Meek (34)	2014	PRK	Texas	67/63	Quinolone antibiotics	Pregabalin/Placebo	4d	VAS, AE
Yong Woo Lee (35)	2014	PRK	South Korea	103/96	Opioid	Fentanyl/Tramadoland + Acetaminophen	3d	AE
Vinicius B. P. Pereira (5)	2017	PRK	Brazil	40/40	Opioid	Codeine0.03% + Acetaminophen0.5%/Placebo	3d	VAS, AE
Mehrdad Mohammadpour (36)	2018	aaPRK	Iran	30/30	NSAIDs	Ketorolac0.5%/ Diclofenac0.1%	4/d for 1 day	VAS
Madeline Ripa (37)	2020	aaPRK	USA	68/67	NSAIDs	Ketorolac0.4%/ Naproxen0.22%	2/d	VAS
Sanwal Javaid (38)	2021	aaPRK	Pakistan	20/20	Opioid	Ketorolac0.4%/ Naproxen0.22%	2/d	VAS
Cherilyn Mae A. Palochak (39)	2021	PRK	Germany	97/100	Opioid	Codeine0.03%/Oxycodone0.005%	4d	VAS
Yuan Wang (40)	2022	Bilateral tPRK	China	51	NSAIDs	Diclofenac0.1%/ Placebo		VAS

T, Intervention group; C, Control group.

### 3.3 Risk of bias in studies

**Figure 2:**
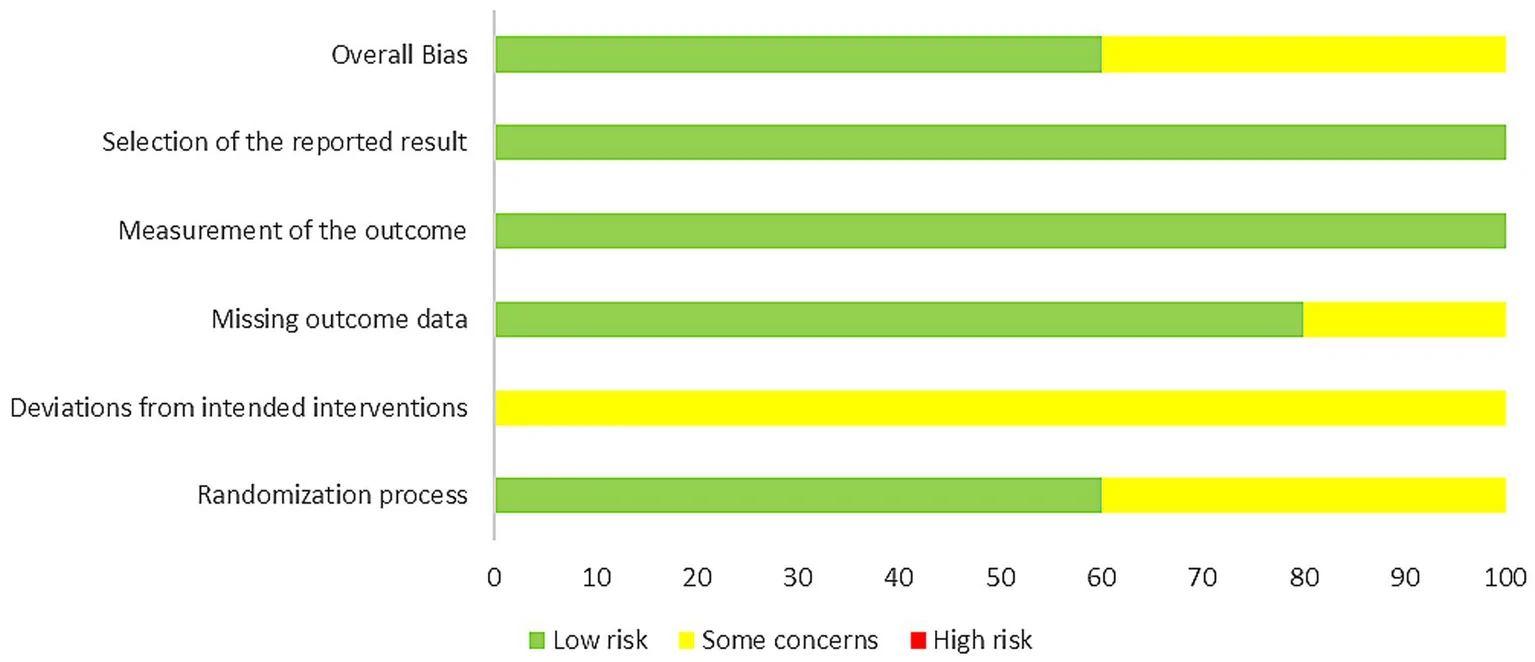
Risk of bias summary of included studies using the RoB 2 tool.

Risk of bias was assessed using the Cochrane RoB 2 tool across five domains. All 28 studies were rated as low risk for the randomization domain. For allocation concealment, 15 studies reported adequate methods and were rated low risk, while 13 studies provided insufficient information to permit a clear judgment (rated as “some concerns”). Regarding blinding of participants and personnel, 21 studies were rated low risk, 5 studies were rated as “some concerns” and 2 studies were rated as high risk. For blinding of outcome assessment, 23 studies were rated low risk, 5 studies were rated as “some concerns” and none were rated high risk. Attrition bias was low in all studies, with complete outcome data reported. Selective reporting was also rated low risk across all studies. For other sources of bias (e.g., baseline imbalance, early termination, or industry funding), 21 studies were rated low risk, while 9 studies were rated as “some concerns” due to insufficient reporting of potential confounding factors. Overall, 13 studies (46.4%) were rated as low risk of bias across all domains, 12 (42.9%) raised some concerns in at least one domain, and 3 (10.7%) were classified as high risk in at least one domain, primarily driven by a lack of blinding (Figure 2).

### 3.4 Meta-analysis

#### 3.4.1 VAS

**Figure 3:**
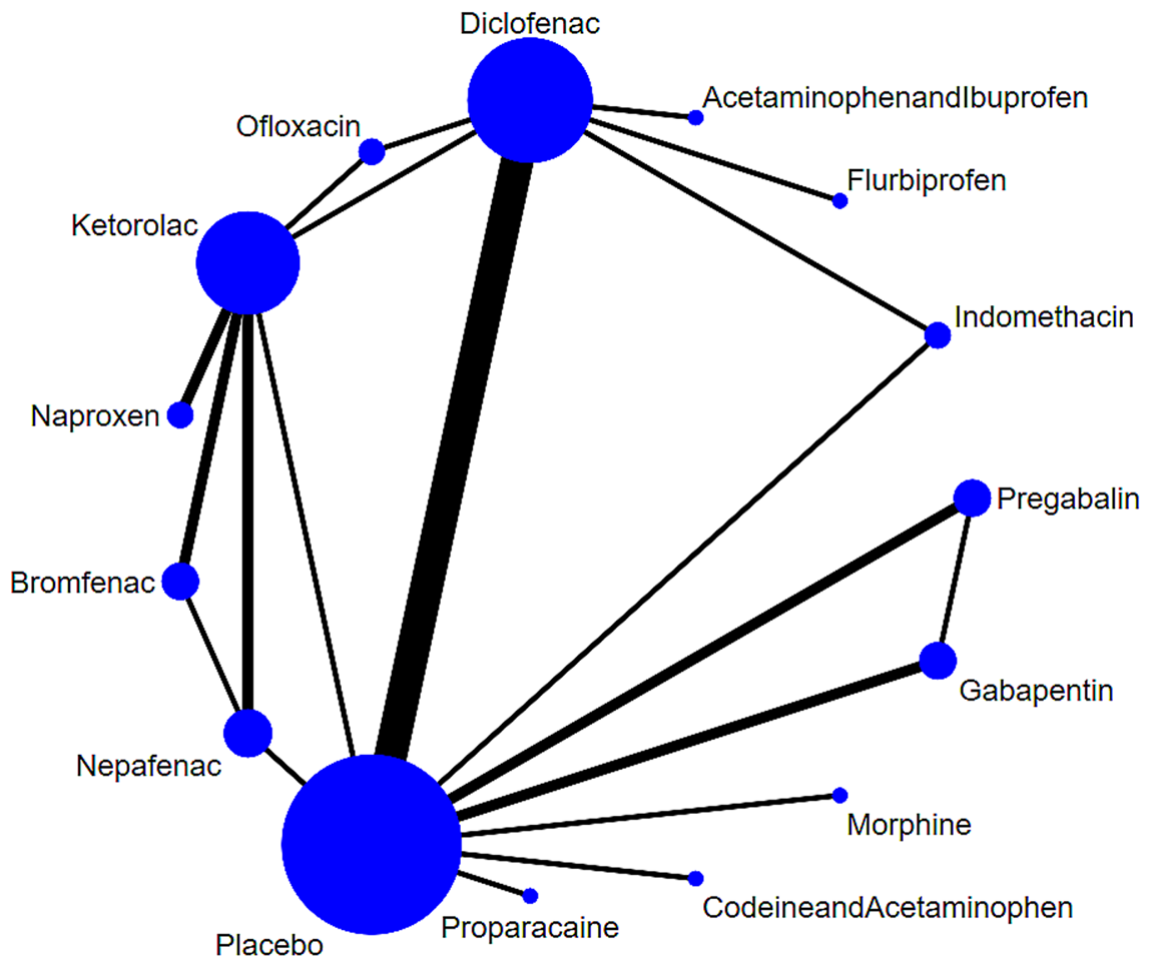
Network plot for the outcome of visual analog scale (VAS). Node size is proportional to the total sample size of each intervention; edge thickness is proportional to the number of direct comparisons between interventions.

**Figure 4:**
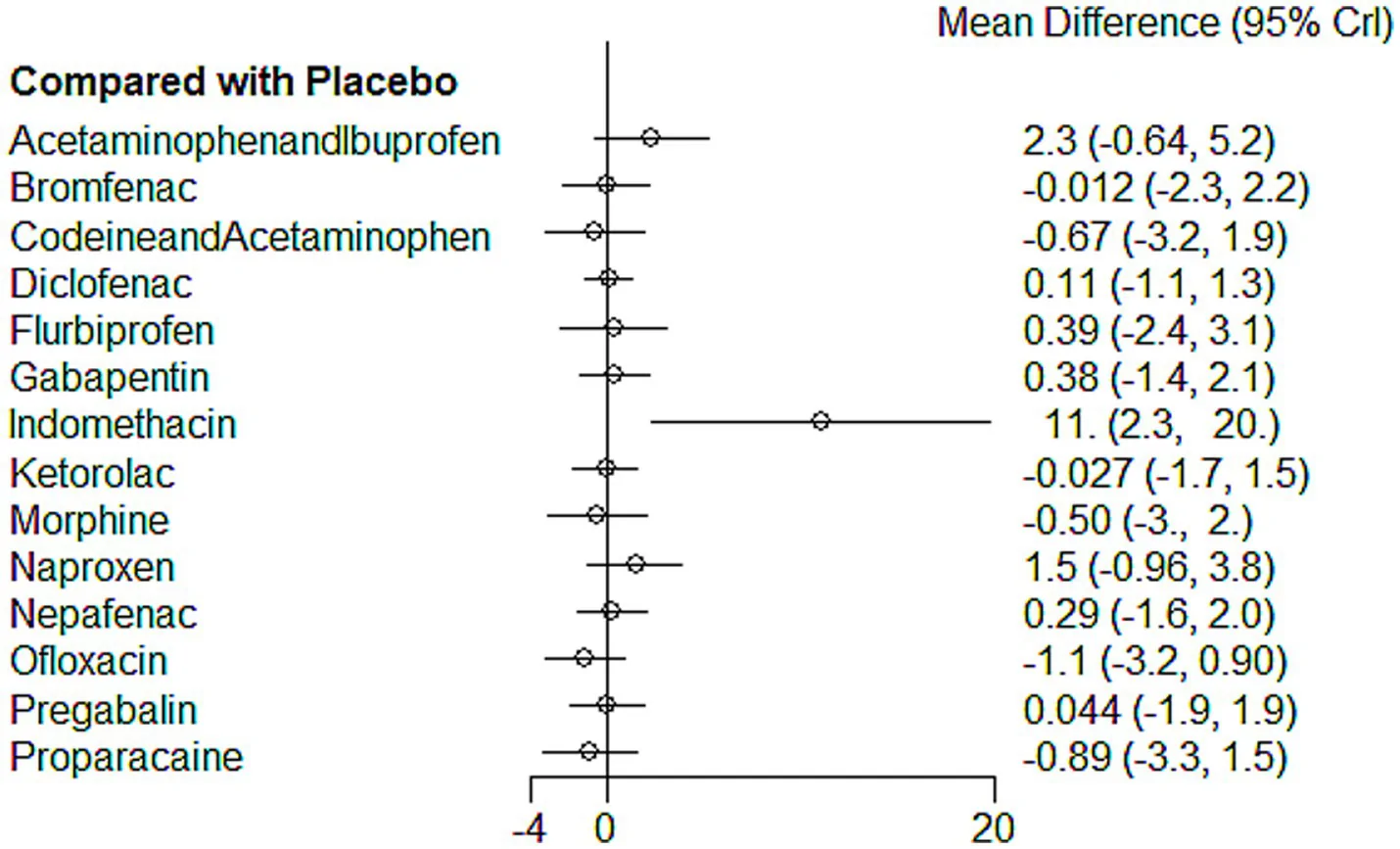
Forest plot for VAS: Mean differences (MD) with 95% credible intervals (CrI) for each intervention compared to placebo. Intervals crossing the null indicate lack of statistical significance.

24 studies involving 14 drugs reported VAS data. The network structure featured closed loops, and inconsistency assessments supported the consistency assumption (all *p* > 0.05 via the node-splitting method; Figure 3), with low global heterogeneity (*I*^2^ = 6%). Compared with placebo, none of the 14 active drugs showed a statistically significant reduction in VAS scores (Figure 4), as evidenced by 95% credible intervals spanning the null value [e.g., Ofloxacin: MD = −1.09, 95% CrI (−3.21, 0.90); Proparacaine: MD = −0.89, 95% CrI (−3.34, 1.52); codeine combined with acetaminophen: MD = −0.67, 95% CrI (−3.18, 1.87)]. Among the 91 pairwise comparisons between active drugs, only two yielded statistically significant differences: Ofloxacin versus Acetaminophen combined with Ibuprofen [MD = −3.41, 95% CrI (−6.65, −0.16)] and Ofloxacin versus Naproxen [MD = −2.57, 95% CrI (−5.11, −0.01)]. The remaining pairwise comparisons did not achieve statistical significance (Supplementary Table S2). Indomethacin was associated with significantly worse outcomes relative to placebo [MD = 10.99, 95% CrI (2.29, 19.66)], a finding derived from a single small-sample trial (*n* = 40). The SUCRA values, which reflect ranking probabilities rather than effect magnitudes, were 0.84 for Ofloxacin, 0.76 for Proparacaine, and 0.71 for Codeine combined with Acetaminophen (Table 2). However, given that no active agent demonstrated clear superiority over placebo, and only 2 of the 91 active-drug comparisons achieved statistical significance—a proportion close to the 5% expected by chance alone—these SUCRA rankings do not constitute evidence of clinical efficacy and should not guide treatment selection. They are presented for descriptive purposes only.

**Table 2:** SUCRA ranking.

Intervention	VAS		Adverse reaction	
	SUCRA	No	SUCRA	No
Acetaminophen + Ibuprofen	0.15	14		
Bromfenac	0.56	6		
Codeine + Acetaminophen	0.71	3	0.18	6
Diclofenac	0.51	9	0.81	1
Flurbiprofen	0.46	11		
Gabapentin	0.44	12		
Indomethacin	0.009	15		
Ketorolac	0.57	5	0.70	2
Morphine	0.67	4		
Naproxen	0.21	13		
Nepafenac	0.466	10	0.53	3
Ofloxacin	0.84	1		
Placebo	0.55	7	0.41	4
Pregabalin	0.54	8	0.34	5
Proparacaine	0.76	2		

#### 3.4.2 Adverse reaction

**Figure 5:**
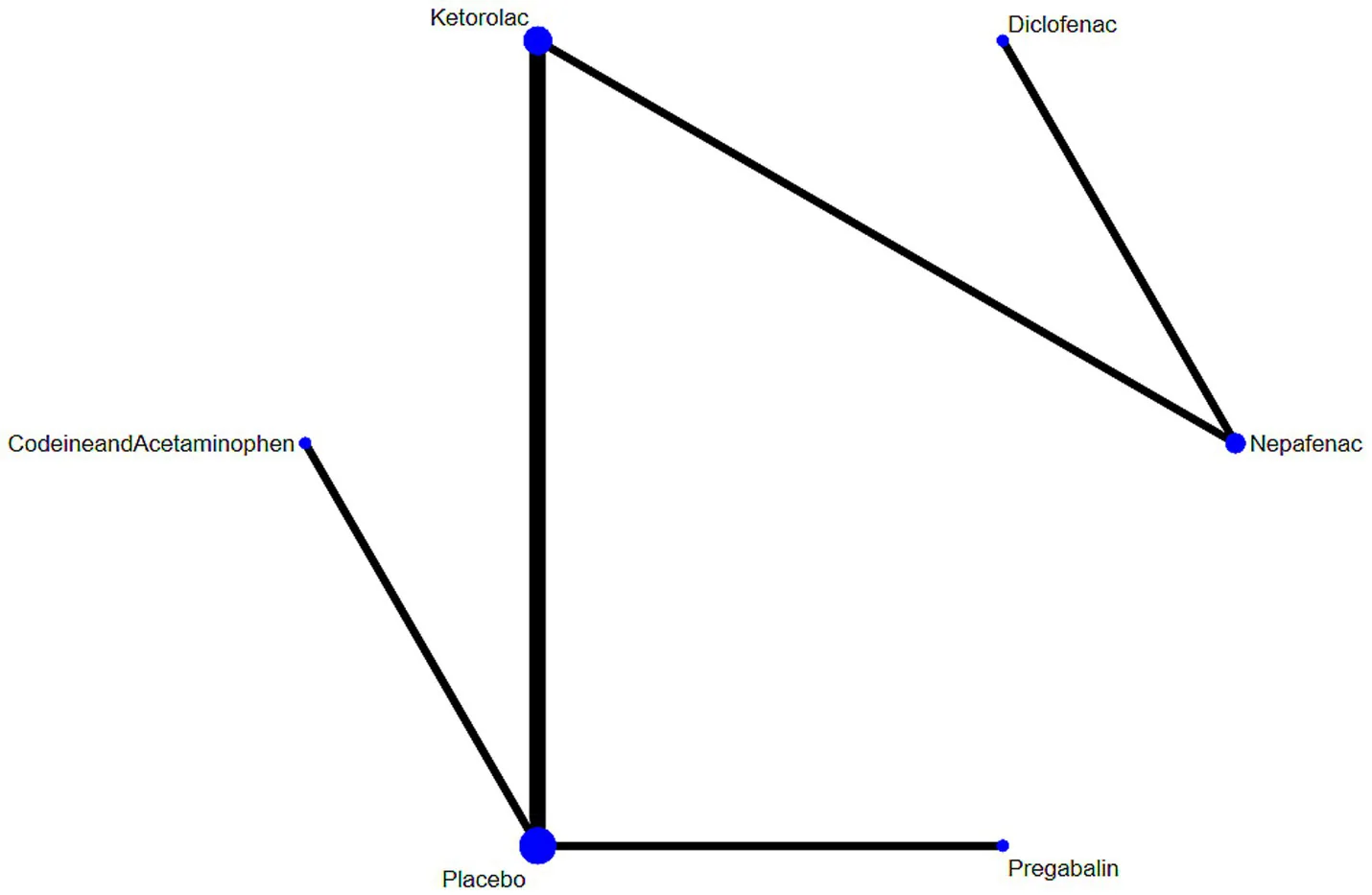
Network plot for adverse events. Node size represents sample size; edge thickness represents the number of direct comparisons.

**Figure 6:**
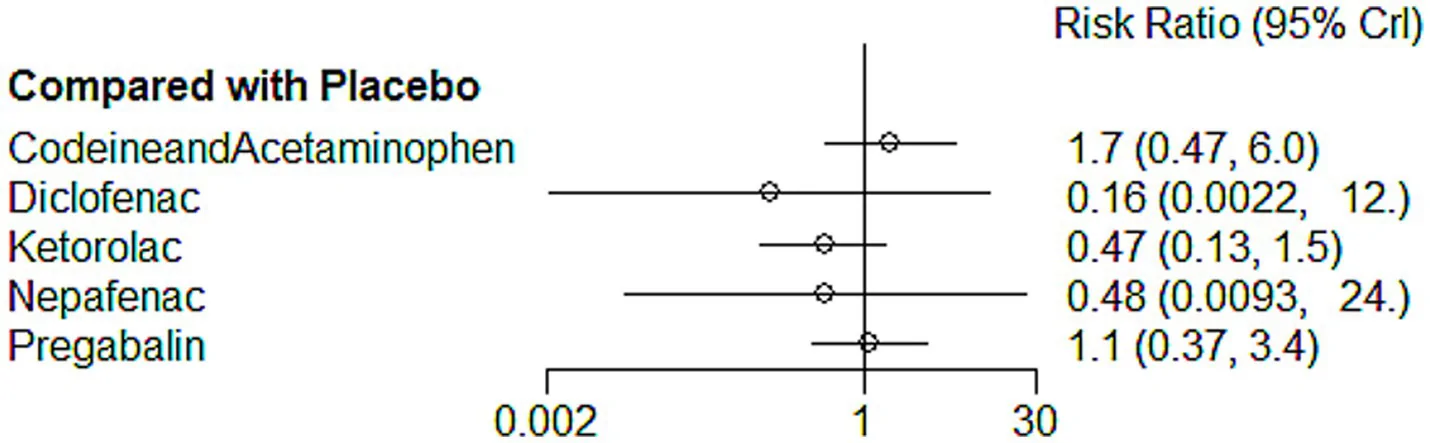
Forest plot for adverse events: relative risks (*RR*) with 95% credible intervals (*CrI*) for each intervention compared to placebo.

Seven studies involving five drugs reported adverse events. The network contained no closed loops, and a consistency model was applied (Figure 5), with low global heterogeneity (*I*^2^ = 7%). Compared with placebo, none of the five drugs demonstrated a statistically significant difference in the risk of adverse events (Figure 6), as evidenced by 95% credible intervals spanning the null value [e.g., Diclofenac: RR = 0.16, 95% CrI (0.002, 11.84); Nepafenac: RR = 0.48, 95% CrI (0.009, 23.79); Ketorolac: RR = 0.47, 95% CrI (0.13, 1.51)]. No statistically significant differences were detected in any of the 15 pairwise comparisons between active drugs, with all 95% CrIs spanning 1 (Supplementary Table S3). The SUCRA values for safety—derived from the network meta-analysis model—were 0.81 for Diclofenac, 0.70 for Ketorolac, and 0.53 for Nepafenac (Table 2). However, given the absence of closed loops (which limits the strength of indirect comparisons), the lack of statistically significant differences relative to placebo, and the extremely wide CrIs for several comparisons [e.g., Diclofenac vs. Placebo: 95% CrI (0.002, 11.84)], these rankings are highly unstable and should not constitute evidence of superior safety. They are presented for descriptive purposes only. Separate from the NMA results, crude adverse event counts—reported without denominators in the original studies, thereby precluding the calculation of incidence rates—were highest for Pregabalin (39 events, reported only as a total count without event-level detail), Fentanyl (20 events), and Codeine combined with Acetaminophen (13 events). The most frequently reported individual adverse events were nausea (16 cases), drowsiness (11 cases), and headache (11 cases) (Table 3). These raw counts are derived from individual study reports and are independent of the network meta-analysis model; therefore, they should not be interpreted as NMA-based rankings. Because denominators were not consistently reported, these raw counts cannot guide safety comparisons between drugs. Furthermore, the extremely wide CrIs observed for Diclofenac and Nepafenac versus placebo reflect the very small number of events in these comparisons, indicating that the effect estimates are highly imprecise and that no meaningful inferences regarding relative safety can be drawn from these data.

**Table 3:** Adverse reaction.

Drug	Number	Adverse reaction
Ketorolac	12	One case of burning sensation, three cases of eye pain, two cases of headache, one case of corneal infiltration, one case of eyelid edema, one case of conjunctival congestion, one case of flu-like symptoms, one case of anxiety, and one case of epithelial defect
Placebo	22	Two cases of conjunctivitis, six cases of eye pain, eight cases of headache, one case of corneal infiltration, two cases of nausea, one case of vomiting, one case of drowsiness, and one case of constipation
Diclofenac	2	One case of influenza syndrome and one case of accidental injury
Nepafenac	1	One case of conjunctivitis
Pregabalin	39	unspecified
Fentanyl	20	Nausea in 14 cases, vomiting in 3 cases, dizziness in 2 cases, and skin rash at the application site in 1 case
Tramadola + Acetaminophen	4	Three cases of abdominal discomfort and one case of diarrhea
Codeine + Acetaminophen	13	Two cases of nausea, one case of headache, and 10 cases of drowsiness

Crude adverse event counts are derived from individual study reports and are independent of the NMA model; they should not be interpreted as NMA-based rankings. Denominators for incidence rates were not consistently reported, precluding meaningful safety comparisons. Pregabalin data were reported only as an aggregate count (39 events) without event-level detail. Fentanyl and Tramadol + Acetaminophen are listed descriptively only; they were not NMA nodes due to lack of suitable comparators for network connectivity. No sight-threatening or life-threatening events were documented. AE, adverse event.

### 3.5 Publication of biased results

**Figure 7:**
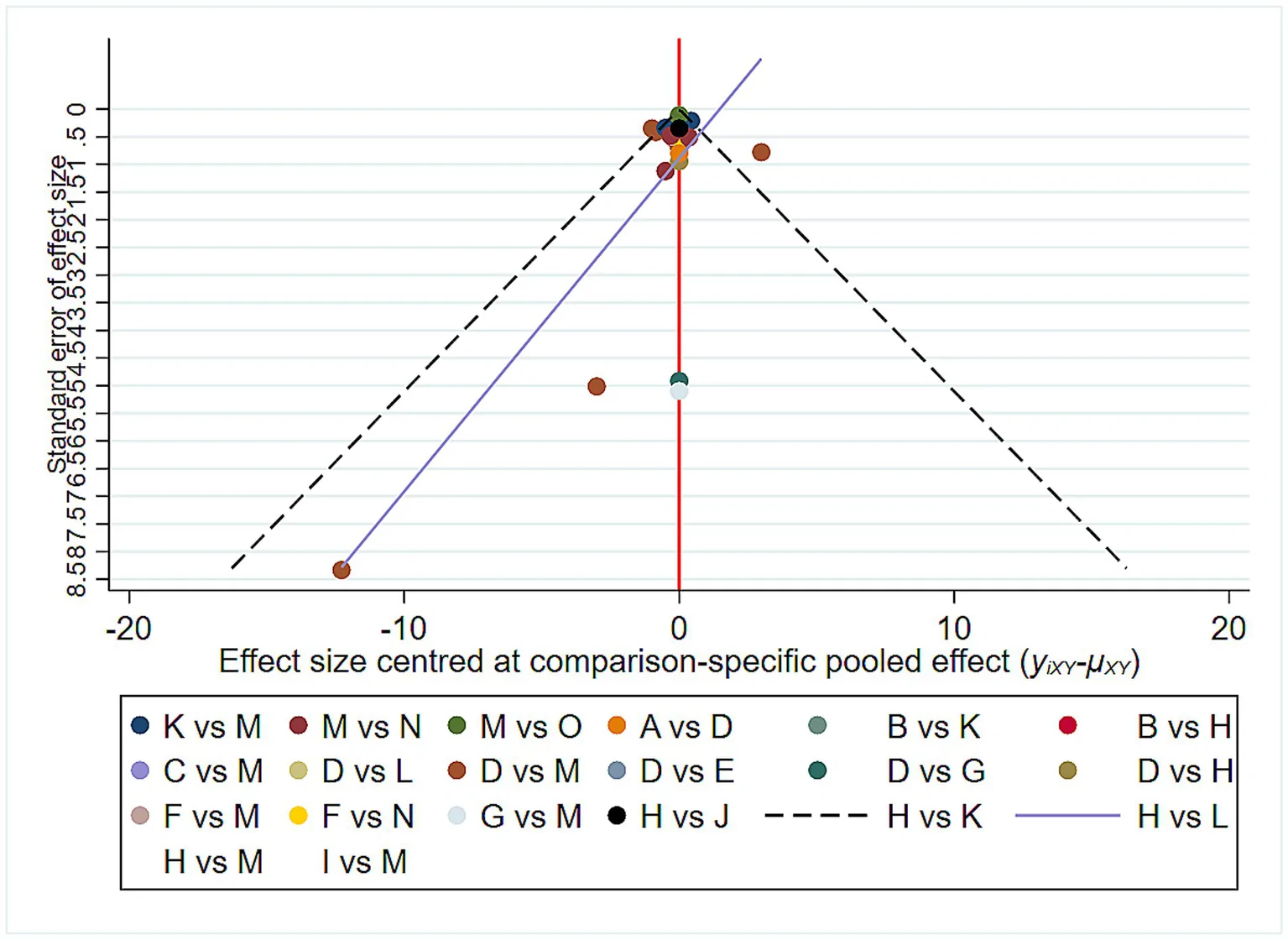
Comparison-adjusted funnel plot for VAS outcome. Egger’s test *p* = 0.74, suggesting low likelihood of publication bias.

Publication bias was assessed using comparison-adjusted funnel plots for the VAS outcome, with Egger’s test yielding a non-significant result (*p* = 0.74), which suggests a low likelihood of small-study effects for this outcome (Figure 7). However, several limitations of this assessment warrant acknowledgment. First, comparison-adjusted funnel plots in network meta-analysis are primarily designed to detect small-study effects rather than definitive publication bias; moreover, their interpretation becomes challenging when the network includes few studies per pairwise comparison. Second, formal publication bias testing was only feasible for the VAS outcome due to the limited number of studies reporting adverse events (*n* = 7), thereby precluding a reliable assessment for safety outcomes. Third, the inclusion of different drug classes with varying levels of regulatory approval and commercial interest may introduce class-specific publication bias that remains undetected by global funnel plot asymmetry tests. Therefore, while Egger’s test does not suggest significant small-study effects for pain outcomes, the possibility of publication bias—particularly regarding null or negative safety findings—cannot be excluded (Figure 7).

### 3.6 Quality of evidence assessment results

The certainty of evidence was assessed using the CINeMA framework for the 10 pairwise comparisons with direct evidence (seven for the VAS outcome, three for adverse events). Of these, two (20%) were rated as high quality, five (50%) as moderate quality, and three (30%) as low quality (Table 4). For the VAS outcome, two comparisons were of high quality (Ketorolac vs. placebo; Proparacaine vs. placebo) and five were of moderate quality, with the latter downgraded for imprecision due to wide CrIs spanning the null value. Notably, the high-quality rating for proparacaine—despite being derived from a single trial—is fully consistent with CINeMA principles; the underlying study was well-designed with a low risk of bias, and the effect estimate was sufficiently precise. CINeMA does not automatically downgrade evidence based on a limited number of trials; rather, imprecision is evaluated by the width of the CrI relative to the minimal important difference, not by study count alone. In contrast, the comparison of Diclofenac versus placebo (comprising five studies) was downgraded to moderate quality due to an imprecise pooled estimate with a wide CrI spanning the null value, reflecting substantial between-study variability. For adverse events, all three direct comparisons were of low quality, downgraded due to both severe imprecision and the inability to assess incoherence owing to the absence of closed loops in the adverse event network. Within-study bias did not warrant downgrading for any comparison.

**Table 4:** CINeMA result.

Intervening measure	Comparison	Number of studies	Within-study bias	Reporting bias	Indirectness	Imprecision	Heterogeneity	Incoherence	Confidence rating	Reason(s) for downgrading
VAS	Codeine and Acetaminophen: Placebo	1	No concerns	Low risk	No concerns	Some concerns	No concerns	No concerns	Moderate	[“Imprecision”]
	Diclofenac: Placebo	5	No concerns	Low risk	No concerns	No concerns	No concerns	No concerns	Moderate	[]
	Gabapentin: Placebo	2	No concerns	Low risk	No concerns	Some concerns	No concerns	No concerns	Moderate	[“Imprecision”]
	Ketorolac: Placebo	1	No concerns	Low risk	No concerns	No concerns	No concerns	No concerns	High	[]
	Morphine: Placebo	1	No concerns	Low risk	No concerns	Some concerns	No concerns	No concerns	Moderate	[“Imprecision”]
	Nepafenac: Placebo	2	No concerns	Low risk	No concerns	Some concerns	No concerns	No concerns	Moderate	[“Imprecision”]
	Placebo: Pregabalin	2	No concerns	Low risk	No concerns	Some concerns	No concerns	No concerns	Moderate	[“Imprecision”]
	Placebo: Proparacaine	1	No concerns	Low risk	No concerns	No concerns	No concerns	No concerns	High	[]
Adverse reaction	Codeine and Acetaminophen: Placebo	1	No concerns	Low risk	No concerns	Some concerns	No concerns	Some concerns	Low	[“Imprecision,”"Incoherence”]
	Ketorolac: Placebo	2	No concerns	Low risk	No concerns	Some concerns	No concerns	Some concerns	Low	[“Imprecision,”"Incoherence”]
	Placebo: Pregabalin	1	No concerns	Low risk	No concerns	Some concerns	No concerns	Some concerns	Low	[“Imprecision,”"Incoherence”]

### 3.7 Sensitivity analysis

A leave-one-out sensitivity analysis was performed to assess the robustness of the VAS network findings. Sequential exclusion of individual studies yielded pooled estimates ranging from −0.147 to 0.121, with all 95% credible intervals spanning the null value, indicating that no single study disproportionately influenced the primary results (Supplementary Tables S1, S2). Notably, when the t-PRK study by Wang et al. (40) was excluded, the pooled estimate shifted minimally from [MD = 0.021, 95% CrI (−0.459, 0.501)] to [MD = −0.047, 95% CrI (−0.543, 0.450)], confirming that the inclusion of this trial did not materially affect the overall conclusions (Supplementary Tables S2, S3).

## 4 Discussion

This network meta-analysis of 28 RCTs (2,797 patients) evaluated 14 drugs across five classes for post-PRK pain management. The primary finding is that no active drug demonstrated a statistically significant advantage over placebo in reducing VAS scores at 24 h post-surgery. Only two of 91 active-drug pairwise comparisons reached nominal significance (Ofloxacin vs. Acetaminophen combined with Ibuprofen and Ofloxacin vs. Naproxen), a proportion close to chance expectation. This pattern—absence of superiority over placebo alongside isolated inter-drug differences—suggests that the observed signals are driven by network sparsity, wide credible intervals, and estimation uncertainty, rather than reflecting true pharmacological superiority. In the context of previous systematic reviews that have largely focused on single drug classes such as NSAIDs (4) or pregabalin (6, 7), the present study offers a more comprehensive comparative paradigm by simultaneously evaluating multiple therapeutic classes within a single network. However, the inherent constraints of the current evidence base—most notably the sparse number of trials contributing to each direct comparison and the substantial reliance on indirect evidence for a majority of the treatment loops—inevitably limits the statistical power and certainty of our network. Consequently, these factors constrain the strength of any definitive comparative conclusions that can be drawn from this analysis.

Regarding safety, no drug differed significantly from placebo in adverse event risk, and none of the 15 active-drug pairwise comparisons reached significance. The AE network lacked closed loops, meaning all active-drug comparisons relied solely on indirect evidence. Crude event counts were reported without denominators in most studies, precluding incidence rate calculation; raw frequency comparisons are not meaningful. Sparse and incomplete safety data preclude firm conclusions, consistent with prior concerns about AE underreporting in post-PRK trials (4). These limitations echo concerns raised in previous reviews: NSAIDs may delay corneal epithelial healing with variable safety profiles across agents (4); opioids are associated with nausea, drowsiness, and constipation (41); local anesthetics carry risks of corneal toxicity with prolonged use (42); and neuromodulators have uncertain long-term effects (7). The present findings do not provide evidence to support the safety superiority of any particular drug or class, and the absence of statistically significant differences should not be interpreted as evidence of equivalent safety given the limited data available.

The efficacy and safety of Diclofenac as a commonly used non-steroidal anti-inflammatory drug (NSAID) in pain management after PRK have been validated by multiple studies (Supplementary Table S4). Previous systematic reviews and meta-analyses have examined the efficacy of specific drug classes for post-PRK pain, but none have simultaneously compared such a broad range of agents within a single analytic framework. A recent systematic review and network meta-analysis by Ben Ephraim Noyman et al. focused exclusively on topical NSAIDs and found significant differences in analgesic efficacy among individual NSAIDs, with flurbiprofen showing the best effect on postoperative days 1–3 (4). That review did not include other drug classes such as opioids, neuromodulators, or antibiotics, and its conclusions were based on a narrower evidence base restricted to NSAIDs. Another systematic review by Chen et al. focused on gabapentin and pregabalin, demonstrating their efficacy in reducing post-refractive surgery pain, but did not compare these agents against other drug classes (6). Similarly, Shen et al. reviewed pregabalin specifically for ocular pain but did not extend the comparison to other classes (7). The present study extends the existing evidence base by providing a comparative assessment across multiple drug classes, thereby offering a more comprehensive perspective. However, the finding that no drug outperformed placebo across the entire network suggests that the comparative advantages suggested by prior class-specific reviews may not translate into meaningful superiority when evaluated against a common placebo reference within a broader comparative framework. An important observation from this analysis warrants explicit discussion. We found that no drug significantly outperformed placebo, yet isolated pairwise comparisons between active drugs showed nominal significance. This apparent paradox requires careful interpretation. Statistically, it is possible for two interventions to show a significant difference between them even when neither is significantly different from placebo, particularly when the placebo comparisons have wider confidence intervals due to smaller sample sizes or greater variability, while the direct head-to-head comparison may be more precise. However, this pattern is more commonly a manifestation of network sparsity, where limited data lead to unstable estimates, and the probability of false-positive findings increases with the number of comparisons made. Given that only two of 91 comparisons reached nominal significance—a proportion close to the 5% expected by chance alone—the most plausible interpretation is that these findings reflect random variation rather than true pharmacological superiority. This observation reinforces the importance of interpreting inter-drug comparisons within the context of the overall network, rather than focusing on isolated pairwise findings in isolation.

This study has several strengths. The systematic search, rigorous adherence to Cochrane methodology, and use of CINeMA for evidence certainty assessment strengthen the reliability of the findings. The inclusion of multiple drug classes within a single analytic framework provides a broader comparative perspective than previous class-specific reviews, and the exploration of both efficacy and safety outcomes offers a more comprehensive assessment. The use of a hierarchical analytic approach—with route-stratified primary analyses and a combined network as sensitivity analysis—provides clinically meaningful comparisons while preserving methodological rigor. However, several methodological limitations warrant emphasis. First, substantial clinical heterogeneity exists across studies (spanning 1996–2022, with variations in drug dosage, timing of administration, and pain assessment time points from 2 h to 7 days), which may affect transitivity. While the reported *I*^2^ values were low (6–7%), this statistical metric should be interpreted with caution in the context of sparse networks. With few studies contributing to each direct comparison, *I*^2^ is known to be an unstable estimate and may substantially underestimate the true degree of heterogeneity due to limited power. Therefore, the absence of statistical heterogeneity does not preclude clinically meaningful heterogeneity, and the findings should be generalized with this limitation in mind. Second, geographic concentration in North America and the Middle East may limit generalizability to populations with different clinical practices or drug access, though this was not formally analyzed as an effect modifier (3, 27, 29, 33, 40). Third, publication bias was testable only for VAS outcomes; publication bias for safety outcomes cannot be excluded, and the small number of studies reporting adverse events further limits the assessment of reporting bias. Fourth, several comparisons suffered from sparse evidence with extremely wide credible intervals [e.g., Diclofenac vs. placebo: RR = 0.16, 95% CrI (0.002, 12)], indicating that many estimates are imprecise and should be interpreted with caution. Fifth, only one t-PRK study was included (40), which may limit generalizability to t-PRK populations; however, a sensitivity analysis excluding this study yielded consistent results with the primary analysis (Supplementary Table S5). This suggests that the inclusion of this study did not materially bias the overall findings, but the limited representation of t-PRK populations remains a consideration for generalizability.

Despite the absence of robust evidence for superiority over placebo, several strategies drawn from the broader literature may help optimize post-PRK pain management while minimizing adverse effects. These include drug-soaked bandage contact lenses for sustained release, which may provide continuous drug delivery and improve patient compliance (3); combination regimens to improve analgesia while reducing individual drug doses, such as the combination of NSAIDs with local anesthetics or opioids (43); and preoperative prophylactic use, such as starting nepafenac two days before surgery (44). For high-risk patients—particularly those with autoimmune conditions, pre-existing ocular surface disease, or a history of chronic pain—individualized assessment and enhanced postoperative monitoring are recommended, as case reports have documented serious corneal complications (e.g., corneal melting) with topical NSAID use in rheumatoid arthritis patients (45). Individualized treatment selection should also consider patient-specific factors such as baseline pain sensitivity, risk of dry eye, and systemic tolerability of opioids or neuromodulators. Future research should prioritize large, well-designed, head-to-head RCTs that compare active drugs directly rather than against placebo alone, as placebo-controlled designs are no longer sufficient to inform clinical decision-making in this context (4, 36, 40). Such trials should incorporate standardized outcome assessment time points (e.g., 24 h post-surgery as the primary endpoint), transparent safety reporting with denominators to enable incidence rate calculation, and greater geographic diversity to enhance the generalizability of findings. Studies specifically addressing t-PRK populations are needed, given the limited representation of this subtype in the current evidence base (40). Furthermore, efforts to identify patient-level predictors of treatment response—such as baseline pain sensitivity, depressive symptoms, corneal healing capacity, and dry eye status—could facilitate more individualized analgesic selection (2). Long-term safety and efficacy outcomes, including impacts on corneal epithelial healing, visual outcomes, and patient satisfaction, should also be prioritized in future research.

## 5 Conclusion

This network meta-analysis demonstrated no evidence that any of the 14 evaluated active drugs provide superior pain relief compared with placebo at 24 h post-PRK. The observed inter-drug differences were minimal and statistically fragile; thus, the SUCRA rankings remain strictly exploratory and should not guide routine clinical practice. Furthermore, available safety data were too sparse and heterogeneous to support meaningful comparative conclusions regarding adverse events. Until more robust evidence emerges, clinicians should continue to rely on established local protocols, individual patient characteristics, and meticulous risk–benefit assessments. Future high-quality, head-to-head randomized controlled trials with standardized outcomes and transparent safety reporting are urgently warranted to inform evidence-based analgesic selection for post-PRK pain management.

## Data Availability

The original contributions presented in the study are included in the article/Supplementary material, further inquiries can be directed to the corresponding author.

## Footnotes

1
https://www.crd.york.ac.uk/PROSPERO/view/CRD420251238092
