# Supplementary material for: Comparison of common pharmacological strategies for post-photorefractive keratectomy pain management: a systematic review and network meta-analysis
Source: Front Med (Lausanne). 2026 Aug 10;13:1904962. doi: 10.3389/fmed.2026.1904962 (PMC13500256; doi:10.3389/fmed.2026.1904962)
Supplement: Supplementary file 1 [file Table_1.DOCX]

***Supplementary Material***

1 **Supplementary Tables**

Table S1 Search strategy

| **Search number** | **Query for PubMed** | **Query for Embase** | **Query for Cochrane Library** | **Query for Web of science** |
| --- | --- | --- | --- | --- |
| 1 | Gabapentin OR Neurontin OR Convalis OR Pregabalin OR 3 isobutyl GABA OR CI1008 OR Lyrica OR Duloxetine Hydrochloride OR Duloxetine OR LY227942 OR LY248686 OR Cymbalta OR Amitriptyline OR Lentizol OR Saroten OR Tryptizol OR Tryptanol OR Elavil OR Laroxyl OR Damilen OR Sarotex OR Tryptine OR Syneudon OR Triptafen OR Novoprotect OR Amineurin OR Amitrip OR Amitrol OR Anapsique OR Domical OR Amitriptylin OR Endep OR Acetaminophen OR Acetominophen OR APAP OR Paracetamol OR Hydroxyacetanilide OR Algotropyl OR Acamol OR Anacin3 OR Datril OR Panadol OR Tylenol OR Acetaco OR Acephen OR Tramadol OR Tramal OR K315 OR Zamudol OR Adolonta OR Contramal OR Amadol OR Biodalgic OR Jutadol OR Nobligan OR Prontofort OR Theradol OR Takadol OR Tiral OR Topalgic OR Tradol OR Tradonal OR Tralgiol OR Trama AbZ OR Trama KD OR TramaDorsch OR Biokanol OR Tramabeta OR Tramadoc OR Ranitidin 1A Pharma OR Trama 1A Pharma OR Zydol OR Ultram OR Tramagetic OR Tramadura OR Tramex OR Tramagit OR Tramake OR Tramadolor OR Tramundin OR Trasedal OR Xymel 50 OR Zumalgic OR Zytram OR Tramadin OR Hydrocodone OR Dihydrocodeinone OR Hydrocodon OR Hydrocon OR Codinovo OR Dicodid OR Hycodan OR Hycon OR Robidone OR Oxycodone OR Dihydrohydroxycodeinone OR Dihydrone OR Oxycodeinon OR Oxycone OR Theocodin OR Eucodal OR Oxiconum OR Pancodine OR Oxycontin OR Dinarkon OR Morphine OR Morphia OR SDZ202250 OR Oramorph SR OR MS Contin OR Duramorph OR Codeine OR N Methylmorphine OR Isocodeine OR Ardinex OR Dexamethasone OR Hexadecadrol OR Methylfluorprednisolone OR Oradexon OR Millicorten OR Hexadrol OR Dexasone OR Decaspray OR Decameth OR Decaject OR Dexpak OR Maxidex OR Clonidine OR Klofenil OR Clofenil OR ST155 OR M5041T OR Klofelin OR Clofelin OR Clopheline OR Isoglaucon OR Hemiton OR Gemiton OR Dixarit OR Chlophazolin OR Catapres[Title/Abstract] | Gabapentin OR Neurontin OR Convalis OR Pregabalin OR 3 isobutyl GABA OR CI1008 OR Lyrica OR Duloxetine Hydrochloride OR Duloxetine OR LY227942 OR LY248686 OR Cymbalta OR Amitriptyline OR Lentizol OR Saroten OR Tryptizol OR Tryptanol OR Elavil OR Laroxyl OR Damilen OR Sarotex OR Tryptine OR Syneudon OR Triptafen OR Novoprotect OR Amineurin OR Amitrip OR Amitrol OR Anapsique OR Domical OR Amitriptylin OR Endep OR Acetaminophen OR Acetominophen OR APAP OR Paracetamol OR Hydroxyacetanilide OR Algotropyl OR Acamol OR Anacin3 OR Datril OR Panadol OR Tylenol OR Acetaco OR Acephen OR Tramadol OR Tramal OR K315 OR Zamudol OR Adolonta OR Contramal OR Amadol OR Biodalgic OR Jutadol OR Nobligan OR Prontofort OR Theradol OR Takadol OR Tiral OR Topalgic OR Tradol OR Tradonal OR Tralgiol OR Trama AbZ OR Trama KD OR TramaDorsch OR Biokanol OR Tramabeta OR Tramadoc OR Ranitidin 1A Pharma OR Trama 1A Pharma OR Zydol OR Ultram OR Tramagetic OR Tramadura OR Tramex OR Tramagit OR Tramake OR Tramadolor OR Tramundin OR Trasedal OR Xymel 50 OR Zumalgic OR Zytram OR Tramadin OR Hydrocodone OR Dihydrocodeinone OR Hydrocodon OR Hydrocon OR Codinovo OR Dicodid OR Hycodan OR Hycon OR Robidone OR Oxycodone OR Dihydrohydroxycodeinone OR Dihydrone OR Oxycodeinon OR Oxycone OR Theocodin OR Eucodal OR Oxiconum OR Pancodine OR Oxycontin OR Dinarkon OR Morphine OR Morphia OR SDZ202250 OR Oramorph SR OR MS Contin OR Duramorph OR Codeine OR N Methylmorphine OR Isocodeine OR Ardinex OR Dexamethasone OR Hexadecadrol OR Methylfluorprednisolone OR Oradexon OR Millicorten OR Hexadrol OR Dexasone OR Decaspray OR Decameth OR Decaject OR Dexpak OR Maxidex OR Clonidine OR Klofenil OR Clofenil OR ST155 OR M5041T OR Klofelin OR Clofelin OR Clopheline OR Isoglaucon OR Hemiton OR Gemiton OR Dixarit OR Chlophazolin OR Catapres[Title/Abstract] | Gabapentin OR Neurontin OR Convalis OR Pregabalin OR 3 isobutyl GABA OR CI1008 OR Lyrica OR Duloxetine Hydrochloride OR Duloxetine OR LY227942 OR LY248686 OR Cymbalta OR Amitriptyline OR Lentizol OR Saroten OR Tryptizol OR Tryptanol OR Elavil OR Laroxyl OR Damilen OR Sarotex OR Tryptine OR Syneudon OR Triptafen OR Novoprotect OR Amineurin OR Amitrip OR Amitrol OR Anapsique OR Domical OR Amitriptylin OR Endep OR Acetaminophen OR Acetominophen OR APAP OR Paracetamol OR Hydroxyacetanilide OR Algotropyl OR Acamol OR Anacin3 OR Datril OR Panadol OR Tylenol OR Acetaco OR Acephen OR Tramadol OR Tramal OR K315 OR Zamudol OR Adolonta OR Contramal OR Amadol OR Biodalgic OR Jutadol OR Nobligan OR Prontofort OR Theradol OR Takadol OR Tiral OR Topalgic OR Tradol OR Tradonal OR Tralgiol OR Trama AbZ OR Trama KD OR TramaDorsch OR Biokanol OR Tramabeta OR Tramadoc OR Ranitidin 1A Pharma OR Trama 1A Pharma OR Zydol OR Ultram OR Tramagetic OR Tramadura OR Tramex OR Tramagit OR Tramake OR Tramadolor OR Tramundin OR Trasedal OR Xymel 50 OR Zumalgic OR Zytram OR Tramadin OR Hydrocodone OR Dihydrocodeinone OR Hydrocodon OR Hydrocon OR Codinovo OR Dicodid OR Hycodan OR Hycon OR Robidone OR Oxycodone OR Dihydrohydroxycodeinone OR Dihydrone OR Oxycodeinon OR Oxycone OR Theocodin OR Eucodal OR Oxiconum OR Pancodine OR Oxycontin OR Dinarkon OR Morphine OR Morphia OR SDZ202250 OR Oramorph SR OR MS Contin OR Duramorph OR Codeine OR N Methylmorphine OR Isocodeine OR Ardinex OR Dexamethasone OR Hexadecadrol OR Methylfluorprednisolone OR Oradexon OR Millicorten OR Hexadrol OR Dexasone OR Decaspray OR Decameth OR Decaject OR Dexpak OR Maxidex OR Clonidine OR Klofenil OR Clofenil OR ST155 OR M5041T OR Klofelin OR Clofelin OR Clopheline OR Isoglaucon OR Hemiton OR Gemiton OR Dixarit OR Chlophazolin OR Catapres[Title/Abstract] | Gabapentin OR Neurontin OR Convalis OR Pregabalin OR 3 isobutyl GABA OR CI1008 OR Lyrica OR Duloxetine Hydrochloride OR Duloxetine OR LY227942 OR LY248686 OR Cymbalta OR Amitriptyline OR Lentizol OR Saroten OR Tryptizol OR Tryptanol OR Elavil OR Laroxyl OR Damilen OR Sarotex OR Tryptine OR Syneudon OR Triptafen OR Novoprotect OR Amineurin OR Amitrip OR Amitrol OR Anapsique OR Domical OR Amitriptylin OR Endep OR Acetaminophen OR Acetominophen OR APAP OR Paracetamol OR Hydroxyacetanilide OR Algotropyl OR Acamol OR Anacin3 OR Datril OR Panadol OR Tylenol OR Acetaco OR Acephen OR Tramadol OR Tramal OR K315 OR Zamudol OR Adolonta OR Contramal OR Amadol OR Biodalgic OR Jutadol OR Nobligan OR Prontofort OR Theradol OR Takadol OR Tiral OR Topalgic OR Tradol OR Tradonal OR Tralgiol OR Trama AbZ OR Trama KD OR TramaDorsch OR Biokanol OR Tramabeta OR Tramadoc OR Ranitidin 1A Pharma OR Trama 1A Pharma OR Zydol OR Ultram OR Tramagetic OR Tramadura OR Tramex OR Tramagit OR Tramake OR Tramadolor OR Tramundin OR Trasedal OR Xymel 50 OR Zumalgic OR Zytram OR Tramadin OR Hydrocodone OR Dihydrocodeinone OR Hydrocodon OR Hydrocon OR Codinovo OR Dicodid OR Hycodan OR Hycon OR Robidone OR Oxycodone OR Dihydrohydroxycodeinone OR Dihydrone OR Oxycodeinon OR Oxycone OR Theocodin OR Eucodal OR Oxiconum OR Pancodine OR Oxycontin OR Dinarkon OR Morphine OR Morphia OR SDZ202250 OR Oramorph SR OR MS Contin OR Duramorph OR Codeine OR N Methylmorphine OR Isocodeine OR Ardinex OR Dexamethasone OR Hexadecadrol OR Methylfluorprednisolone OR Oradexon OR Millicorten OR Hexadrol OR Dexasone OR Decaspray OR Decameth OR Decaject OR Dexpak OR Maxidex OR Clonidine OR Klofenil OR Clofenil OR ST155 OR M5041T OR Klofelin OR Clofelin OR Clopheline OR Isoglaucon OR Hemiton OR Gemiton OR Dixarit OR Chlophazolin OR Catapres[Topic] |
| 2 | Refractive Surgical Procedure* OR Refractive Surgery OR Refractive Surgeries OR Keratorefractive Surgical Procedure* OR Photorefractive Keratectomy OR Photorefractive Keratectomies OR Corneal Surgery, Laser OR Laser Corneal Surgeries OR Laser Corneal Surgery OR Laser Keratectomies OR Photokeratectomy OR Photokeratectomies OR Laser Keratectomy OR Keratectomy, Subepithelial, Laser-Assisted OR Laser Subepithelial Keratomileusis OR LASEK OR Laser-Assisted Subepithelial Keratomileusis OR Subepithelial Photorefractive Keratectomy OR Subepithelial Photorefractive Keratectomies OR Laser-Assisted Subepithelial Keratectomies OR Laser Assisted Subepithelial Keratectomy  [Title/Abstract] | Refractive Surgical Procedure* OR Refractive Surgery OR Refractive Surgeries OR Keratorefractive Surgical Procedure* OR Photorefractive Keratectomy OR Photorefractive Keratectomies OR Corneal Surgery, Laser OR Laser Corneal Surgeries OR Laser Corneal Surgery OR Laser Keratectomies OR Photokeratectomy OR Photokeratectomies OR Laser Keratectomy OR Keratectomy, Subepithelial, Laser-Assisted OR Laser Subepithelial Keratomileusis OR LASEK OR Laser-Assisted Subepithelial Keratomileusis OR Subepithelial Photorefractive Keratectomy OR Subepithelial Photorefractive Keratectomies OR Laser-Assisted Subepithelial Keratectomies OR Laser Assisted Subepithelial Keratectomy  [Title/Abstract] | Refractive Surgical Procedure* OR Refractive Surgery OR Refractive Surgeries OR Keratorefractive Surgical Procedure* OR Photorefractive Keratectomy OR Photorefractive Keratectomies OR Corneal Surgery, Laser OR Laser Corneal Surgeries OR Laser Corneal Surgery OR Laser Keratectomies OR Photokeratectomy OR Photokeratectomies OR Laser Keratectomy OR Keratectomy, Subepithelial, Laser-Assisted OR Laser Subepithelial Keratomileusis OR LASEK OR Laser-Assisted Subepithelial Keratomileusis OR Subepithelial Photorefractive Keratectomy OR Subepithelial Photorefractive Keratectomies OR Laser-Assisted Subepithelial Keratectomies OR Laser Assisted Subepithelial Keratectomy  [Title/Abstract] | Refractive Surgical Procedure* OR Refractive Surgery OR Refractive Surgeries OR Keratorefractive Surgical Procedure* OR Photorefractive Keratectomy OR Photorefractive Keratectomies OR Corneal Surgery, Laser OR Laser Corneal Surgeries OR Laser Corneal Surgery OR Laser Keratectomies OR Photokeratectomy OR Photokeratectomies OR Laser Keratectomy OR Keratectomy, Subepithelial, Laser-Assisted OR Laser Subepithelial Keratomileusis OR LASEK OR Laser-Assisted Subepithelial Keratomileusis OR Subepithelial Photorefractive Keratectomy OR Subepithelial Photorefractive Keratectomies OR Laser-Assisted Subepithelial Keratectomies OR Laser Assisted Subepithelial Keratectomy  [Topic] |
| 3 | Pain OR Physical Suffering* OR Ache*[All Fields] | Pain OR Physical Suffering* OR Ache*[All Fields] | Pain OR Physical Suffering* OR Ache*[All Fields] | Pain OR Physical Suffering* OR Ache*[All Fields] |
| 4 | Randomised OR Randomized OR Blind OR phase OR multicentre OR control[Title/Abstract] | Randomised OR Randomized OR Blind OR phase OR multicentre OR control[Title/Abstract] | Randomised OR Randomized OR Blind OR phase OR multicentre OR control[Title/Abstract] | Randomised OR Randomized OR Blind OR phase OR multicentre OR control[Title/Abstract] |
| 5 | 1 AND 2 AND 3 AND 4 | 1 AND 2 AND 3 AND 4 | 1 AND 2 AND 3 AND 4 | 1 AND 2 AND 3 AND 4 |

Table S2 VAS

| Acetaminophen and Ibuprofen | Bromfenac | Codeine and Acetaminophen | Diclofenac | Flurbiprofen | Gabapentin | Indomethacin | Ketorolac | Morphine | Naproxen | Nepafenac | Ofloxacin | Placebo | Pregabalin | Proparacaine |
| --- | --- | --- | --- | --- | --- | --- | --- | --- | --- | --- | --- | --- | --- | --- |
| Acetaminophen and Ibuprofen | -2.33 (-5.83, 1.16) | -2.98 (-6.75, 0.91) | -2.2 (-4.85, 0.44) | -1.93 (-5.53, 1.71) | -1.93 (-5.27, 1.49) | 8.69 (-0.41, 17.73) | -2.35 (-5.46, 0.76) | -2.82 (-6.58, 1.05) | -0.83 (-4.41, 2.71) | -2.03 (-5.31, 1.25) | -3.41 (-6.65, -0.16) | -2.31 (-5.15, 0.64) | -2.27 (-5.72, 1.21) | -3.21 (-6.95, 0.61) |
| 2.33 (-1.16, 5.83) | Bromfenac | -0.65 (-3.95, 2.77) | 0.13 (-2.16, 2.42) | 0.4 (-2.95, 3.79) | 0.39 (-2.38, 3.29) | 11.01 (2.08, 19.91) | -0.01 (-1.73, 1.67) | -0.49 (-3.77, 2.92) | 1.49 (-0.94, 3.89) | 0.3 (-1.69, 2.3) | -1.08 (-3.59, 1.42) | 0.01 (-2.18, 2.3) | 0.06 (-2.85, 3.03) | -0.88 (-4.12, 2.47) |
| 2.98 (-0.91, 6.75) | 0.65 (-2.77, 3.95) | Codeine and Acetaminophen | 0.78 (-2.06, 3.51) | 1.06 (-2.72, 4.72) | 1.06 (-2.04, 4.11) | 11.65 (2.61, 20.66) | 0.64 (-2.43, 3.56) | 0.17 (-3.39, 3.69) | 2.15 (-1.39, 5.52) | 0.96 (-2.2, 3.98) | -0.42 (-3.76, 2.76) | 0.67 (-1.87, 3.18) | 0.71 (-2.51, 3.86) | -0.23 (-3.74, 3.26) |
| 2.2 (-0.44, 4.85) | -0.13 (-2.42, 2.16) | -0.78 (-3.51, 2.06) | Diclofenac | 0.28 (-2.19, 2.75) | 0.27 (-1.79, 2.41) | 10.9 (2.19, 19.55) | -0.14 (-1.81, 1.49) | -0.62 (-3.34, 2.18) | 1.36 (-1.05, 3.73) | 0.17 (-1.78, 2.09) | -1.2 (-3.12, 0.67) | -0.11 (-1.25, 1.1) | -0.07 (-2.3, 2.2) | -1.01 (-3.68, 1.74) |
| 1.93 (-1.71, 5.53) | -0.4 (-3.79, 2.95) | -1.06 (-4.72, 2.72) | -0.28 (-2.75, 2.19) | Flurbiprofen | -0.01 (-3.23, 3.29) | 10.61 (1.61, 19.6) | -0.42 (-3.41, 2.53) | -0.9 (-4.55, 2.85) | 1.09 (-2.38, 4.48) | -0.1 (-3.27, 3.01) | -1.49 (-4.63, 1.62) | -0.39 (-3.09, 2.38) | -0.35 (-3.68, 3.02) | -1.29 (-4.92, 2.42) |
| 1.93 (-1.49, 5.27) | -0.39 (-3.29, 2.38) | -1.06 (-4.11, 2.04) | -0.27 (-2.41, 1.79) | 0.01 (-3.29, 3.23) | Gabapentin | 10.61 (1.73, 19.44) | -0.41 (-2.85, 1.89) | -0.89 (-3.92, 2.16) | 1.1 (-1.92, 3.96) | -0.1 (-2.66, 2.35) | -1.47 (-4.22, 1.16) | -0.38 (-2.14, 1.37) | -0.34 (-2.53, 1.81) | -1.28 (-4.27, 1.71) |
| -8.69 (-17.73, 0.41) | -11.01 (-19.91, -2.08) | -11.65 (-20.66, -2.61) | -10.9 (-19.55, -2.19) | -10.61 (-19.6, -1.61) | -10.61 (-19.44, -1.73) | Indomethacin | -11.03 (-19.81, -2.26) | -11.48 (-20.49, -2.47) | -9.53 (-18.5, -0.58) | -10.72 (-19.56, -1.88) | -12.1 (-20.95, -3.22) | -10.99 (-19.66, -2.29) | -10.94 (-19.84, -2.02) | -11.88 (-20.87, -2.86) |
| 2.35 (-0.76, 5.46) | 0.01 (-1.67, 1.73) | -0.64 (-3.56, 2.43) | 0.14 (-1.49, 1.81) | 0.42 (-2.53, 3.41) | 0.41 (-1.89, 2.85) | 11.03 (2.26, 19.81) | Ketorolac | -0.48 (-3.37, 2.56) | 1.51 (-0.23, 3.23) | 0.31 (-1.2, 1.85) | -1.07 (-2.94, 0.83) | 0.03 (-1.52, 1.72) | 0.07 (-2.38, 2.61) | -0.88 (-3.73, 2.13) |
| 2.82 (-1.05, 6.58) | 0.49 (-2.92, 3.77) | -0.17 (-3.69, 3.39) | 0.62 (-2.18, 3.34) | 0.9 (-2.85, 4.55) | 0.89 (-2.16, 3.92) | 11.48 (2.47, 20.49) | 0.48 (-2.56, 3.37) | Morphine | 1.98 (-1.53, 5.33) | 0.79 (-2.35, 3.79) | -0.59 (-3.88, 2.56) | 0.5 (-1.99, 3) | 0.55 (-2.63, 3.67) | -0.4 (-3.87, 3.08) |
| 0.83 (-2.71, 4.41) | -1.49 (-3.89, 0.94) | -2.15 (-5.52, 1.39) | -1.36 (-3.73, 1.05) | -1.09 (-4.48, 2.38) | -1.1 (-3.96, 1.92) | 9.53 (0.58, 18.5) | -1.51 (-3.23, 0.23) | -1.98 (-5.33, 1.53) | Naproxen | -1.19 (-3.47, 1.13) | -2.57 (-5.11, -0.01) | -1.48 (-3.77, 0.96) | -1.44 (-4.42, 1.66) | -2.38 (-5.7, 1.09) |
| 2.03 (-1.25, 5.31) | -0.3 (-2.3, 1.69) | -0.96 (-3.98, 2.2) | -0.17 (-2.09, 1.78) | 0.1 (-3.01, 3.27) | 0.1 (-2.35, 2.66) | 10.72 (1.88, 19.56) | -0.31 (-1.85, 1.2) | -0.79 (-3.79, 2.35) | 1.19 (-1.13, 3.47) | Nepafenac | -1.38 (-3.67, 0.91) | -0.29 (-2.02, 1.55) | -0.24 (-2.82, 2.4) | -1.18 (-4.15, 1.87) |
| 3.41 (0.16, 6.65) | 1.08 (-1.42, 3.59) | 0.42 (-2.76, 3.76) | 1.2 (-0.67, 3.12) | 1.49 (-1.62, 4.63) | 1.47 (-1.16, 4.22) | 12.1 (3.22, 20.95) | 1.07 (-0.83, 2.94) | 0.59 (-2.56, 3.88) | 2.57 (0.01, 5.11) | 1.38 (-0.91, 3.67) | Ofloxacin | 1.09 (-0.9, 3.21) | 1.14 (-1.64, 3.98) | 0.19 (-2.94, 3.43) |
| 2.31 (-0.64, 5.15) | -0.01 (-2.3, 2.18) | -0.67 (-3.18, 1.87) | 0.11 (-1.1, 1.25) | 0.39 (-2.38, 3.09) | 0.38 (-1.37, 2.14) | 10.99 (2.29, 19.66) | -0.03 (-1.72, 1.52) | -0.5 (-3, 1.99) | 1.48 (-0.96, 3.77) | 0.29 (-1.55, 2.02) | -1.09 (-3.21, 0.9) | Placebo | 0.04 (-1.9, 1.95) | -0.89 (-3.34, 1.52) |
| 2.27 (-1.21, 5.72) | -0.06 (-3.03, 2.85) | -0.71 (-3.86, 2.51) | 0.07 (-2.2, 2.3) | 0.35 (-3.02, 3.68) | 0.34 (-1.81, 2.53) | 10.94 (2.02, 19.84) | -0.07 (-2.61, 2.38) | -0.55 (-3.67, 2.63) | 1.44 (-1.66, 4.42) | 0.24 (-2.4, 2.82) | -1.14 (-3.98, 1.64) | -0.04 (-1.95, 1.9) | Pregabalin | -0.94 (-4.02, 2.17) |
| 3.21 (-0.61, 6.95) | 0.88 (-2.47, 4.12) | 0.23 (-3.26, 3.74) | 1.01 (-1.74, 3.68) | 1.29 (-2.42, 4.92) | 1.28 (-1.71, 4.27) | 11.88 (2.86, 20.87) | 0.88 (-2.13, 3.73) | 0.4 (-3.08, 3.87) | 2.38 (-1.09, 5.7) | 1.18 (-1.87, 4.15) | -0.19 (-3.43, 2.94) | 0.89 (-1.52, 3.34) | 0.94 (-2.17, 4.02) | Proparacaine |

Table S3 Adverse reaction

| Codeine and Acetaminophen | Diclofenac | Ketorolac | Nepafenac | Placebo | Pregabalin |
| --- | --- | --- | --- | --- | --- |
| Codeine and Acetaminophen | 0.1 (0, 8.59) | 0.28 (0.05, 1.58) | 0.29 (0, 17.52) | 0.6 (0.17, 2.14) | 0.67 (0.12, 3.6) |
| 10.09 (0.12, 902.1) | Diclofenac | 2.81 (0.05, 183.87) | 2.79 (0.48, 24.06) | 6.09 (0.08, 463.04) | 6.77 (0.08, 587.35) |
| 3.52 (0.63, 21.92) | 0.36 (0.01, 21.52) | Ketorolac | 1.05 (0.02, 44.31) | 2.12 (0.66, 7.48) | 2.35 (0.5, 12.59) |
| 3.44 (0.06, 210.7) | 0.36 (0.04, 2.08) | 0.95 (0.02, 40.94) | Nepafenac | 2.07 (0.04, 107.26) | 2.28 (0.04, 140.68) |
| 1.66 (0.47, 6.04) | 0.16 (0, 11.84) | 0.47 (0.13, 1.51) | 0.48 (0.01, 23.79) | Placebo | 1.11 (0.37, 3.4) |
| 1.5 (0.28, 8.03) | 0.15 (0, 12.1) | 0.43 (0.08, 2.01) | 0.44 (0.01, 24.87) | 0.9 (0.29, 2.72) | Pregabalin |

Table S4 Comparison of the advantages and disadvantages as well as approval status of commonly used pain-relieving drugs after PRK surgeries

| **Drug category** | **Representing drugs** | **Efficacy advantage** | **Safety disadvantages/precautions** | **Differences in approval status among countries** |
| --- | --- | --- | --- | --- |
| NSAIDs (Non-steroidal anti-inflammatory) | Ketorolac, diclofenac, flurbiprofen, bromofenac, neparfenamide, indomethacin | Effective inhibition of prostaglandin synthesis, definite analgesic effect. Ketorolac and flurbiprofen showed significant analgesic effects in the early postoperative period (POD0-1). Diclofenac can reduce postoperative foreign body sensation. | Delayed corneal epithelial healing is the most common adverse reaction of this type of drug. Flurbiprofen has a lesser effect on healing, but all NSAIDs should be used with caution. A rare but serious risk of corneal ablation has been reported in patients with rheumatoid arthritis, among others. | Most NSAIDs are approved in major markets such as the United States, the European Union, and China for postoperative inflammation and pain. But specific dosage forms (such as diclofenac soaked bandage endoscopes) may not be approved in all regions. |
| Local anesthetics | Procaine, tetracaine | It takes effect quickly, provides immediate pain relief, and is often used for acute pain management during and after surgery. | There is a high risk of corneal epithelial toxicity, which can significantly delay epithelial healing. Tetracaine causes more severe pain during eye drops and may have a higher risk of delayed healing. It is not recommended for routine long-term use for postoperative analgesia. | Procaine and tetracaine are widely used local anesthetics in various countries, but their use as routine postoperative analgesics is off-label in many countries, including China, as they may delay wound healing. |
| Opioid drugs | Codeine, oxycodone, fentanyl | [Strong analgesic effect, especially for moderate to severe pain. Codeine combined with acetaminophen can significantly reduce pain scores within 72 hours after surgery.](https://www.tandfonline.com/doi/full/10.2147/OPTH.S66701) | [There are many systemic adverse reactions such as nausea, vomiting, drowsiness, constipation, and addiction risk. Fentanyl transdermal patches are convenient to use, but respiratory depression should be watched out for.](https://www.tandfonline.com/doi/full/10.2147/OPTH.S66701) | It is a controlled drug and its approval and use are strictly restricted in various countries. Different countries have different regulations on prescription authority, dosage forms that can be prescribed and courses of treatment. |
| Neuromodulators | Pregabalin, Gabapentin | [By modulating calcium channels to reduce pain signaling, it works for neuropathic pain. Studies have shown that it can lower acute pain scores after surgery.](https://journals.lww.com/jcrs/abstract/2024/10000/topical_nonsteroidal_anti_inflammatory_drugs_for.15.aspx) | [A high incidence of central nervous system adverse reactions (dizziness, drowsiness). Pregabalin had the highest number of adverse events in some studies.](https://www.tandfonline.com/doi/full/10.2147/OPTH.S66701) | Approved for neuropathic pain in major markets such as the United States, the European Union, and China. But for the indication of acute pain after PRK surgery, it is off-label in many countries, including China. |
| Antibiotics (fluoroquinolones) | Ofloxacin | [Dual effects: Preventing bacterial infections while exerting anti-inflammatory and analgesic effects by inhibiting COX-2 expression.](https://pubmed.ncbi.nlm.nih.gov/25263042/) | Its analgesic effect is relatively weak and it is usually not used as a first-line analgesic. Beware of bacterial resistance when used for a long time. | [As an antibiotic, it is widely approved in countries around the world. But its "analgesic" indication has not been officially approved in any country, and it is off-label use based on research findings that it has anti-inflammatory effects.](https://pubmed.ncbi.nlm.nih.gov/25263042/) |
| Glucocorticoids | Dexamethasone, flunomide | [It has strong anti-inflammatory properties and can effectively inhibit postoperative inflammatory responses. It is often used in combination with NSAIDs to reduce the risk of corneal complications.](https://www.mdpi.com/2077-0383/11/14/4109) | Long-term use may increase intraocular pressure, induce cataracts, and delay infection. Beware of fungal infection when using with corneal epithelial defect. | As an anti-inflammatory drug, it is widely approved in various countries. But specific postoperative regimens in combination with NSAIDs may be empirical regimens at each medical center. |

Table S5 Leave-one-out sensitivity analysis for VAS network

| **Study omitted** | **Effect estimate (95% CI)** |
| --- | --- |
| Alireza Eslampoor | 0.007 (-0.486, 0.500) |
| Alireza Eslampour | -0.071 (-0.559, 0.416) |
| Appiotti | 0.036 (-0.462, 0.534) |
| Daniel S. Durrie | 0.039 (-0.454, 0.532) |
| Ella G. Faktorovich | 0.048 (-0.446, 0.542) |
| Eric D. Donnenfeld | 0.008 (-0.488, 0.504) |
| Hassan Razmju | 0.121 (-0.357, 0.600) |
| Hassan Razmju (2) | 0.104 (-0.378, 0.586) |
| Jin Pyo Hong | -0.053 (-0.567, 0.461) |
| Jin Pyo Hong (2) | -0.046 (-0.585, 0.492) |
| Julie M. Meek | 0.013 (-0.474, 0.501) |
| Lee Shahinian | -0.032 (-0.546, 0.481) |
| Madeline Ripa | 0.077 (-0.408, 0.563) |
| Matthew Caldwell | -0.023 (-0.525, 0.479) |
| Matthew D. Kuhnle | 0.006 (-0.493, 0.505) |
| Mehrdad Mohammadpour | 0.056 (-0.431, 0.543) |
| Mehrdad Mohammadpour (2) | -0.037 (-0.533, 0.459) |
| Michael Assouline | 0.045 (-0.434, 0.523) |
| Miles K. Tutton | 0.011 (-0.469, 0.490) |
| Mohammad Pakravan | 0.007 (-0.488, 0.503) |
| Neal A. Sher | 0.025 (-0.480, 0.530) |
| Sanwal Javaid | 0.177 (-0.207, 0.561) |
| Vinicius B. P. Pereira | 0.055 (-0.436, 0.547) |
| Combined (all studies) | 0.021 (-0.459, 0.501) |
| Wang et al. excluded | -0.047 (-0.543, 0.450) |
